# Intimate Partner Violence Perpetration and the Five-Factor Model of Personality: A Systematic Review

**DOI:** 10.1177/15248380241299431

**Published:** 2024-11-27

**Authors:** Elena Dorling, Hauwa Onifade, Kevin Browne

**Affiliations:** 1 University of Nottingham, UK

**Keywords:** intimate partner violence, personality traits, five-factor model, domestic abuse, intimate terrorism, common couple violence

## Abstract

Intimate Partner Violence (IPV) is a global concern that has a large impact on both victims and society. Understanding factors that contribute to the perpetration of IPV can help prevent harm. Personality disorders are largely related to IPV perpetration according to recent research; however, there is a large amount of overlap between different personality disorders, and it has been suggested that personality traits may provide a clearer picture on the aspects of personality that result in IPV. Personality traits develop during childhood, and, despite being largely stable, can be modified through intervention. A systematic review was carried out by searching three large databases, examining personality traits from the Five-Factor Model, the prevailing personality model, and IPV perpetration. Eleven studies were included in the final analysis, largely from community samples. The results suggested that neuroticism demonstrates a significant relationship with the perpetration of IPV. There were some differences between community and forensic studies; however, these could be explained by exploring the different types of IPV in line with Johnson’s distinction between common couple violence and intimate terrorism. Intimate terrorism, which is more likely to be displayed by clinical samples, is less likely to be emotionally motivated and therefore may not be linked to neuroticism. Limitations to the method used in the review and the impact of these on the findings are discussed.

## Introduction

Intimate Partner Violence (IPV) is behavior that causes psychological, sexual, or physical harm that is perpetrated by a current or former intimate partner. It is a global concern, affecting over a quarter of women worldwide (World Health Organization, 2021). IPV does not only impact the victim but has a large cost to society. The British Government estimated that “the social and economic cost for victims of domestic abuse” in 2017 was around £66 billion, with around £14 billion relating to costs for “lost output,” such as absences from work (Home Office, 2019).

Understanding the factors that contribute to the perpetration of IPV is essential in order to prevent abuse and the associated harm. Previous systematic reviews have investigated different factors associated with IPV perpetration, including attachment (Velotti et al., 2018), early maladaptive schemas (Pilkington et al., 2021), and personality disorders (Collison & Lynam, 2021).

### Personality

Personality traits are “dimensions of individual differences in tendencies to show consistent patterns of thoughts, feelings, and actions” (McCrae & Costa, 2002, p. 25). Emotional, interpersonal, experiential, attitudinal, and motivational styles are described by personality traits, and these are suggested to be largely a result of genetic influences (McCrae & Costa, 2002). Personality traits develop during childhood and are thought to be largely stable throughout adulthood, although small changes have been observed after the brain has stopped developing (McCrae & Costa, 2002). However, recent evidence suggests that personality traits are dynamic and can change as a result of interventions (Roberts et al., 2017). Personality traits can influence decisions and actions that individuals make in the experiences and situations they are faced with throughout life (McCrae & Costa, 2002).

Evidence suggests personality disorders can be effectively understood as maladaptive or extreme variations of personality traits (Widiger, 2011). Personality disorders are characterized by problems with cognition, emotional experience and expression, and maladaptive behaviors, which persist for an extended period of time (World Health Organization, 2022). The review by Collison and Lynam (2021) found that most categories of personality disorder were related to IPV perpetration; however, they argued that the degree of overlap between the symptoms of different personality disorders could suggest this is not a particularly useful observation. Many people diagnosed with a personality disorder do not fit into solely one category (Kim & Tyrer, 2010). Instead, investigating personality traits individually may provide a clearer picture on the aspects of personality that could lead to violence within relationships (Collison & Lynam, 2021).

### Five-Factor Model

The Five-Factor Model (FFM) stands as the prevailing framework for understanding the structure of personality (Widiger & Crego, 2019). Costa and McCrae expanded on the work of Eysenck, who identified two initial factors of “neuroticism” and “extraversion,” to identify five broad personality trait dimensions commonly measured in personality inventories (McCrae & John, 1992), which became known as the FFM. The five trait dimensions are openness, conscientiousness, extraversion, agreeableness, and neuroticism.

The “neuroticism” dimension is related to the likelihood of individuals experiencing distress. Individuals high on the neuroticism scale are more likely to experience low self-esteem, irrational thinking, poor impulse control, and poor coping, leading to depression, frustration, guilt, and self-consciousness (McCrae & John, 1992). Extraversion, as opposed to introversion, reflects the degree to which a person actively seeks out and engages in social activities and social interactions. Individuals scoring high on the extraversion scale are more outgoing and assertive than low scorers (Costa & McCrae, 1992). The “Openness to Experience” scale is related to creativity, imagination, and curiosity. High scorers are marked by their vivid imagination, their appreciation for art and beauty, and their preference for novelty over routine (Soto & Jackson, 2013). Agreeableness reflects the degree to which an individual is soft-hearted, generous, good-natured, and lenient (Costa & McCrae, 1992). The final dimension is conscientiousness. High scorers in this domain are hard-working and task-focused, as opposed to the disorganized and distractible presentation of low scorers (Soto & Jackson, 2013). Scores from a measure of the five personality dimensions are reported to correlate highly with scores on the Millon Clinical Multiaxial Inventory, which is a widely used tool for personality assessment (Costa & McCrae, 1990).

The FFM has effectively served as a framework for understanding several personality disorders (Gudonis et al., 2008). Further, there has been a shift toward dimensional models of personality disorders in official diagnostic tools, with a FFM in line with the FFM included in the American Psychiatric Association’s (APA, 2013) Diagnostic and Statistical Manual (Bagby & Widiger, 2018). The FFM is argued to be universal, with research demonstrating consistency across 26 different cultures (McCrae & Costa, 2002). Gender differences across the 26 cultures were also replicated, with men scoring higher on assertiveness, while women scored higher on neuroticism and agreeableness (McCrae & Costa, 2002). However, a study by Gurven et al. (2013) with Tsimane forager-horticulturalist people in Bolivia suggests that the FFM does not fit every population group or culture on Earth.

### FFM and Offending

Different profiles of traits on the FFM show associations with a variety of behaviors and events in life, including well-being, employment, social situations, and criminality (Bagby & Widiger, 2018). Eysenck (1996) argues that personality is the mediating factor between environmental and genetic factors that lead to criminal behavior. Personality traits can also offer some explanation as to why exposure to criminogenic risks factors can produce different results in different individuals (Jones et al., 2011). Personality traits have shown strong correlations to a variety of anti-social and criminal behaviors (Jones et al., 2011). Higher levels of proactive, reactive, and relational aggression are demonstrated by individuals who score lower on the Agreeableness dimension (Miller et al., 2012). Research based on the FFM has shown that anti-social behavior has been linked to low levels of agreeableness and conscientiousness (Vize et al., 2019). Offending behavior has also consistently been linked to these same two trait dimensions, while a low level of openness also demonstrates a connection (Wiebe, 2004). However, in terms of IPV, there is not a clear picture on related personality traits (Collison & Lynam, 2021).

### Aims and Objectives

Maladaptive personality traits, such as those present in Personality Disorders, are argued to be extreme presentations of the FFM, and measures have been developed to capture this (Bagby & Widiger, 2018). Identifying specific personality traits that are linked to IPV could assist with developing effective offending behavior programs. The aim of this review is to identify the personality traits identified by the FFM that are related to IPV. The objective is to determine whether IPV perpetrators display different personality traits, measured according to the FFM dimensions when compared with a comparison group of non-perpetrators and/or general violence offenders in cross-sectional studies.

## Methods

### Inclusion Criteria

To be eligible for inclusion, studies were required to compare the scores of one or more of the personality trait dimensions from the FFM (openness, conscientiousness, extraversion, agreeableness, and neuroticism) between people who perpetrated IPV and those who did not (see Table 1).

**Table 1.** PECO Criteria.

Criteria		Inclusion Criteria	Exclusion Criteria
Population	Adults	Adults of any gender	Younger than 18 years
Exposure	IPV perpetration	Convicted of IPV perpetration Self-report IPV perpetration	Perpetrated solely other types of violence (not against partners) IPV victimization only
Comparison	Non-exposed groups OR Different IPV Types	Non-perpetrators of IPV Measure norms Different types of IPV (psychological, physical, sexual)	No comparison group AND only one IPV type
Outcomes	Personality traits	Personality assessment measuring FFM traits, that is, agreeableness, openness, conscientiousness, extraversion and/or neuroticism	Scores not presented quantitatively Not FFM model traits of personality

*Note.* IPV = Intimate Partner Violence; FFM = Five-Factor Model.

IPV perpetration could be measured by self-report methods or offense histories. IPV perpetrators could be compared with non-IPV offenders or general population comparison groups or measure norms. Studies with males or females, or both, were eligible for inclusion. Studies were excluded if they did not measure traits from the FFM, did not examine the perpetration of IPV (e.g., studying victimization), or the participants were stated to be younger than 18 years.

### Sources of Literature

Three bibliographic databases were searched on the August 28, 2023; PsychINFO with the search covering the time period from 1806 to the date of the search, MEDLINE covering the time period from 1946 to the search date, and Science Direct (search period unknown). These databases produced both published journal articles and gray literature, such as dissertations and PhD theses. These databases covered journals including “Partner Abuse,” “Interpersonal Violence,” “Psychology of Violence,” and “Violence Against Women” (APA, 2023). Once searches were complete and relevant articles identified, the reference lists of included studies were examined to identify any other articles.

### Search Strategy

Search terms were developed on the two concepts: “intimate partner violence” and “personality traits.” The specific search terms used are displayed in Table 2, with an asterisk symbolizing a truncated term and a question mark to capture different spellings to ensure all variations were covered.

**Table 2.** Search Terms.

Concept	Search Terms
IPV	- intimate partner violence - domestic abuse - dating violence - domestic violence - family violence - spousal violence - spousal abuse - domestic batter* - dating abuse - partner agrees* -partner abuse
Personality traits	- personality traits - extr?ver* - agreeable* - openness - conscientious* - neurotic* - big five personality - five-factor model -five-factor personality model

*Note.* IPV = Intimate Partner Violence.

### Study Selection

Studies were screened initially by title, then by abstract. The remaining studies were read in full and included or excluded based on the previously stated criteria.

### Bias/Quality Assessment

To assess the quality of the studies, the Joanne Briggs Institute Checklist for Analytical Cross-Sectional Studies (Moola et al., 2017) was utilized. The quality assessment considered the objectivity and standardization of the measurement tool used to capture both personality traits and IPV perpetration, as well as the clear specification of the sample used. The studies were rated using the tool by the primary author. A second reviewer independently carried out an assessment with the same tool, and the results were compared. Upon comparison of the results, there was disagreement on the inclusion of one article (study 14, see Table 3) and this was discussed by the reviewers. Upon discussion around the sample and the lack of specificity on the measurement of IPV perpetration, as well as the replication of the data set, it was decided to exclude the study.

**Table 3.** Results of Quality Assessment (*N* = 14).

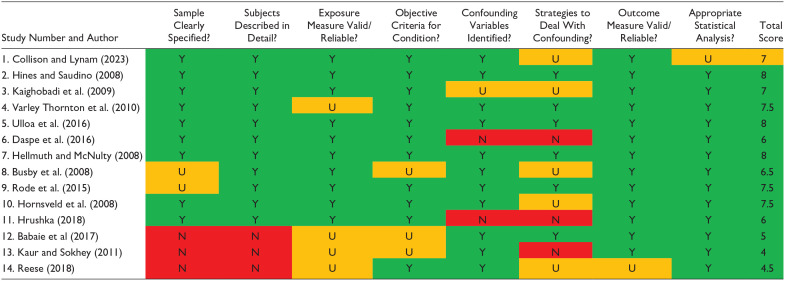

*Note*. Excluded studies in red. Max total score = 8. Y (green) = Yes; N (red) = No; U (amber) = Unclear; n/a = not applicable.

## Results

### Description of Studies

**Figure 1.**
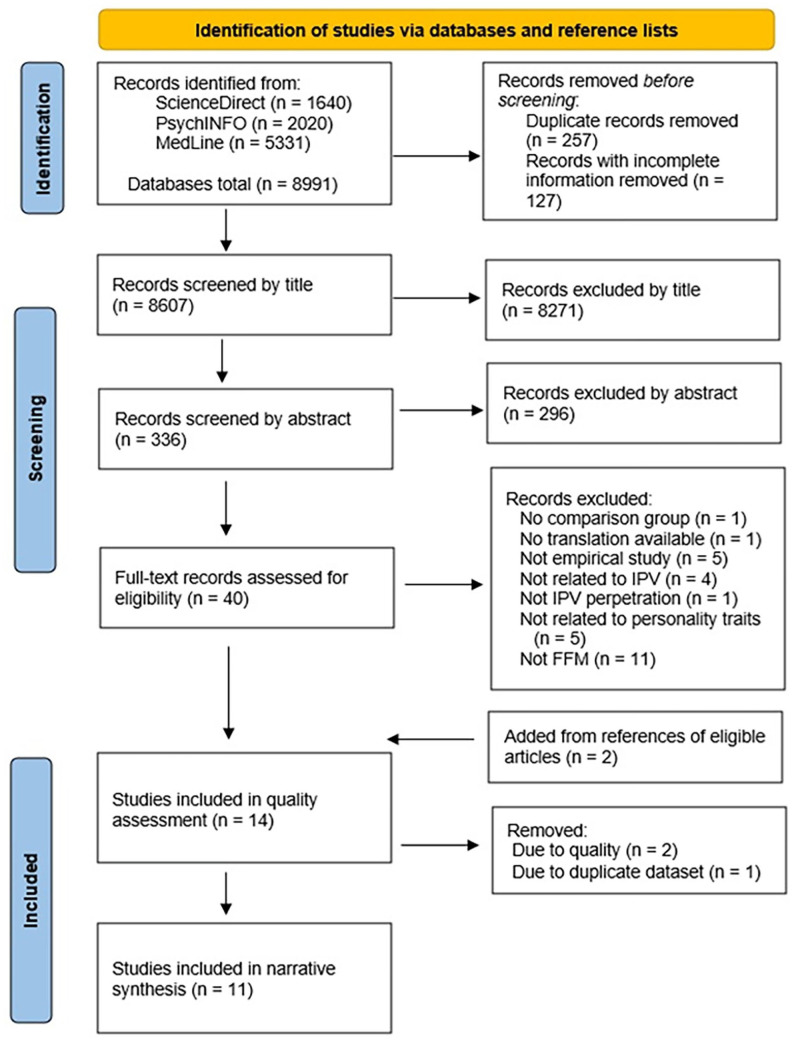
PRISMA flowchart of review process.

The searches produced 8,891 hits, and this was screened down to 40 articles for full-text assessment. The stages of the review process are displayed in Figure 1. Following this, 28 articles were removed, leaving 12 for inclusion. Two additional articles were included following a search of the reference lists for included articles, leaving 14 for quality assessment.

### Quality Assessment Results

The quality assessment results from the Joanna Briggs Institute tool are indicated in Table 3, with the excluded studies marked in red. Two studies were excluded following the quality assessment, leaving a total of 12 studies for inclusion. The study by Babaie et al. (2017) was excluded as the IPV perpetrators were not clearly identified in the sample, and Kaur and Sokhey’s (2011) study was removed as it was unclear how IPV perpetration was measured. To protect against bias, as advised by Cochrane (Higgins et al., 2023), the study by Reese (2018) was not included as it used the same dataset as Ulloa et al. (2016) study but did not provide as much detail about the sample or the methods used to measure the exposure condition.

### Characteristics of Included Studies

The descriptive data of the included studies is presented in Table 4. Three of the studies (9, 10, and 11) examined a forensic sample, while the remaining studies sampled the general population. A total of 40,722 participants (44.62% male) were included in the review, with less than 1% coming from forensic/clinical samples. The general population and forensic sample studies’ results are discussed separately below.

**Table 4.** Descriptive Information for Included Studies (*N* = 11).

Study	Sample Source and Location	Sample Details No. of Ps (no. of males) Mean age, Ethnicity	IPV Measure	Personality Measure	Statistical Analysis Used
1	Online platform, USA	- 307 (139) - M = 39.42 - 82.1% White	CTS2	IPIP-NEO (Maples et al., 2014)—short form	Correlation
2	Colleges, USA	- 480 (179) - M = 19.1 - 77% white	CTS2	IPIP; (Goldberg, 1999) and EPI (Eysenck & Eysenck, 1975)	Regression
3	University and Community, USA	- 467 (467) - M = 24.2 - NR	The Violence Assessment Index (Dobash et al., 1995)	Botwin et al.’s (1997) personality tool	Correlation
4	University, UK	- 297 (116) - M = 23.83 - NR	Violent and Non-violent Offending Behavior Scale	IPIP	Correlation and regression
5	National Longitudinal Study of Adolescent Health (Wave 4), USA	- 7,187 (2,876) - M = 29.12 - 61% white	Original CTS (three items)	Mini-IPIP (Donnellan et al., 2006)	Correlation and regression
6	Community, USA	- 598 (279), - Male M = 30.08 Female M = 28.02 - NR	Revised CTS (sexual coercion)	NEO-FFI	Correlation and path analysis
7	Community, NR	- 338 (169) - Male M = 25.6, Female M = 23.4 - Males = 94% white, Females = 86% white	CTS (physical scale)	IPIP	Regression
8	Community, NR	- 30,600 (13,770) - M = 28.5 - 86% white	A question asking them how often their partner was violent toward them	RELATE (Holman et al., 1997)	Pathway analysis
9	Court, Poland	- 227 (122) - M = 36.92 - NR	Criminal convictions/ charges	NEO-FFI	*t*-Tests
10	Outpatients, Netherlands	- 166 (166^a^) - IPV M = 37.32, GV M = 28.88 - NR	Court orders/convictions	NEO-FFI	*t*-Tests and Ancova
11	Therapy groups, Canada	- 55 (55) - NR - 61.8% white	IPV therapy attendance	NEO-FFI-3	*t*-Test

*Note*. NR = not reported; P = participant; IPV = Intimate Partner Violence; M = mean; SD = standard deviation; GV = general violence; IPIP = International Personality Item Pool; EPI = Eysenck Personality Inventory; CTS = Conflict Tactics Scale; Ne = Neuroticism; Ag = agreeableness; C = conscientiousness; Ex = extraversion; ES = emotional stability; OtE = openness to experience.

a Gender not specified but assumed male due to comparison to male norms

* *p* < .01. **p < .05. ***p < .001.

Five of the eight general population studies measured IPV using a form of the Conflict Tactics Scale (CTS). Studies 1 and 2 used the Revised Conflict Tactics Scale (CTS2; Straus et al., 1996), while study 5 used a modified version of the original CTS (Straus, 1979), which contained three items assessing perpetration and three assessing victimization. Study 7 used eight items from the original CTS. Study 6 used a short form of the CTS2 (Straus & Douglas, 2004) which consisted of two items, and also added a question from the original CTS, to measure sexual coercion and aggression.

Study 3 used The Violence Assessment Index and Injury Assessment Index, both developed by Dobash et al. (1995). Study 4 used the Violent and Nonviolent Offending Behavior Scale, developed by one of the authors of the study (Thornton) using items from established tools, but is unpublished, meaning the validity and reliability cannot be independently assessed. Similarly, study 8 used a question that asked how often their partner was physically violent toward them, as opposed to a validated scale. However, they were included as the method of measurement was clearly defined and explained. Five studies (one, two, four, five, and seven) used the International Personality Item Pool (Goldberg, 1999) in some form to measure personality traits. Four studies (6, 9, 10, and 11) used a version of the NEO Five-Factor Inventory (Costa & McCrae, 1992), while study 8 used the Relationship Evaluation Questionnaire (Busby et al., 2001). Study 3 opted for a method used by Botwin et al. (1997) in which participants rate themselves on 40 pairs of bipolar adjectives. The reliability and validity of this method is unknown.

### Descriptive Data Synthesis

Out of the 11 studies included in the analysis, five were carried out in the United States of America (one, two three, five, six), and one each in Canada, the United Kingdom, Poland, and the Netherlands. Two studies (seven and eight) do not state which country the study was located in. Eight studies recruited both male and female participants; however, three studies appear to have male-only samples (3, 10, and 11). It should be noted, however, that study 10 does not explicitly state the sample is male, but the authors compare scores to male norms. Study 1 included both males and females together in their analysis, while studies two, four, five, seven, eight, and nine examined the genders separately. Study 6 surveyed both males and females, although the study only examined male-perpetrated violence.

Five studies measured physical IPV perpetration only (see Table 5). One study measured physical and psychological IPV perpetration; one study looked only at sexual aggression; two studies looked at all three types; and two studies (both forensic samples based on convictions) did not identify the type of IPV. Seven studies included all five personality traits.

**Table 5.** The IPV Types and Personality Traits Explored in Each Study.

Study	IPV Type				Personality Traits				
	Physical	Psych.	Sexual	Not Specified	O	C	E	A	N
1	✓	✓			✓	✓	✓	✓	✓
2	✓	✓	✓		✓	✓	✓	✓	✓
3	✓					✓		✓	✓
4	✓				✓	✓	✓	✓	✓
5	✓				✓	✓	✓	✓	✓
6			✓						✓
7	✓								✓
8	✓								✓
9				✓	✓	✓	✓	✓	✓
10				✓	✓	✓	✓	✓	✓
11	✓	✓	✓		✓	✓	✓	✓	✓

*Note.* IPV = Intimate Partner Violence.

Study 3 examined three of the traits. Study 3 used the term “emotional stability” for “neuroticism,” meaning high emotional stability scores represented low levels of neuroticism, and vice versa. Three studies (six, seven, and eight) looked solely at neuroticism.

### Prevalence of IPV

Of the eight studies that utilized samples from the general population, six reported on the prevalence of violence in relationships. A total of 15% of the sample of Collinson’s study reported perpetrating physical IPV in the previous year. In study 2, 25% of participants reported using physical aggression toward their partner. Study 7 reported that in 4 years, 100% of couples reported using at least one type of aggressive act, 85% reported committing at least four types and 41.4% reported perpetrating all of the eight aggressive acts listed.

Studies 4 and 5 reported on the mean number of perpetrated IPV acts in the previous 12 months. Study 4 reported a mean of 2.91 acts per female and 0.91 per male participant, with study 5 reporting 0.32 for both genders combined. Study 4 also reported on general violence offending, with a mean number of general violence offenses of 7.74 for men and 4.33 for women, suggesting women are more violent in relationships, while men are more violent outside of relationships. Two studies (two and six) reported the prevalence of sexual aggression in relationships. In study 2, 13.2% of females and 29.1% of males reported perpetration, while 19% of participants in study six reported perpetration.

Two studies reported on psychological aggression prevalence. Studies 1 and 2 found similar rates, with 79.4% and 80% of participants, respectively, reporting perpetration. Thus, it appears that psychological aggression is widespread within relationships in the general population.

### Overall Findings

Table 6 displays a breakdown of the significant results found for each personality trait as well as displaying the numbers of the studies who found a relationship by IPV type.

**Table 6.** The Numbers of the Studies Which Found a Relationship Between Each Trait and IPV Type, With the Total Number of Studies, by Direction, Demonstrating a Relationship Between Each Personality Trait and Any Type of IPV, Percentage of the Studies That Examined That Trait That Found A Result in Brackets.

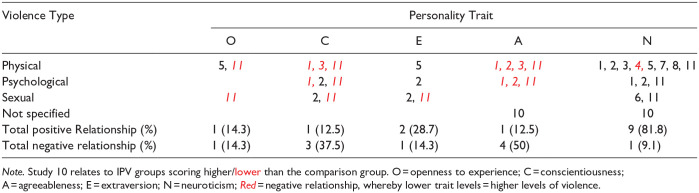

*Note.* Study 10 relates to IPV groups scoring higher/lower than the comparison group. O = openness to experience; C = conscientiousness; A = agreeableness; E = extraversion; N = neuroticism; *Red* = negative relationship, whereby lower trait levels = higher levels of violence.

Two studies found results for the trait “Openness to Experience”; however, the results were in opposition. This could be because the studies sampled different population types (forensic vs general population). This pattern was also present in relation to the trait of extraversion, where two general population studies (two and five) found a positive relationship while a forensic study found a negative relationship.

Half of the studies that examined the personality trait “conscientiousness” found significant results; however, these also illustrated opposing findings. This may be related to the gender of the participants. Three studies found negative relationships. Two of these studies results related to male samples, while the third (study 1) used a mixed-gender sample. On the other hand, study 2 found a positive relationship between psychological IPV and conscientiousness, when perpetrated by females.

The trait of agreeableness ranked second in terms of the number of results indicating a relationship with IPV perpetration, with five of eight studies reporting a significant finding. Three of these were general population studies which all found negative relationships. The remaining two were forensic studies. Study 11’s results were in line with the general population studies; however, study 10 reported a positive relationship. This was likely due to the comparison group used by study 10, who compared IPV perpetrators to generally violent offenders, finding that IPV perpetrators were more agreeable. If compared to a general population group, it may show that non-perpetrators are more agreeable than both groups; however, the study does not examine this.

The trait with the highest number of significant results was neuroticism. However, it is worth noting that neuroticism was the only personality trait explored in all 11 studies. A total of 90.9% of studies found a result relating to neuroticism, with 81.8% showing a positive relationship between neuroticism and IPV perpetration. This included two forensic samples and also covered all three types of IPV, as well as both genders. It is noted, however, that study 2 only found a link between *severe* physical IPV, consisting of the most extreme items on the CTS2 physical scale, and neuroticism in female perpetrators. Conversely, study 4 found a negative relationship for female perpetrators of physical IPV perpetration. This could be related to the use of an unpublished IPV measurement tool, leading to questionable validity. The results from general population studies for each IPV type are described further below.

### General Population Studies

#### Personality Traits—Physical Violence

In total, seven general population studies examined physical aggression. Of these, most studies found a link between neuroticism and violent perpetration. Studies 1 and 5 reported positive correlations between physical IPV perpetration and neuroticism in mixed-gender analyses. Study 3 identified a negative correlation between emotional stability and physical IPV for males. While all the previous studies suggested increased neuroticism (reduced emotional stability) leads to increased perpetration, study 4, on the other hand, reported a negative correlation for females between neuroticism and perpetration. However, it is noted that the correlation coefficients for physical IPV and neuroticism reported by studies one, three, four, and five ranged between −.23 and .18, suggesting the relationship is negligible (Mukaka, 2012).

Using regression analyses, studies 2 and 7 found physical IPV perpetration was linked to high neuroticism in males, and studies 5 and 7 found the same for female perpetrators. Study 2 reported that only severe physical abuse by females was predicted by neuroticism. Study 8 found a link between neuroticism and relationship aggression for both males and females, but it was described as on the “threshold of relevance,” as the relationships were extremely weak.

Three studies found significant results for physical IPV perpetration and the traits of agreeableness and conscientiousness. Studies 1 and 3 reported negative correlations for both traits, although the relationships were weak. Study 2 found low agreeableness predicted physical aggression by females; however, study 4 found that although general violence was negatively linked with agreeableness in women, IPV was not related in any way. Study 5 found higher levels of openness were related to higher levels of IPV perpetration in both genders, while higher levels of extraversion also predicted physical IPV perpetration in women.

### Personality Traits—Sexual Aggression

Two general population studies looked at sexual aggression within relationships. Study 6 found that sexual coercion reported by females was positively correlated with the male partner’s level of neuroticism, although the relationship was weak. However, there was not a significant correlation between neuroticism and male self-reports of the perpetration of sexual coercion. A path analysis from study 6 reported a curvilinear relationship, where at minimal levels of neuroticism, sexual aggression, and neuroticism are negatively related; however, as the level of neuroticism increases to a specific point, the relationship changes and the two demonstrate a positive association.

Study 2 did not find any personality traits correlated/associated with male sexual aggression. Instead, they found that female use of sexual aggression was predicted by higher levels of extraversion and conscientiousness.

### Personality Traits—Psychological Violence

Two general population studies (one and two) examined psychological IPV perpetration, and both found significant results relating to neuroticism and agreeableness. In relation to neuroticism, study 1 found a positive correlation in a mixed-gender sample, and regressions for both male and female participants in study 2 found perpetration was predicted by higher scores. For agreeableness, study 1 reported a negative correlation and study 2 found perpetration was predicted by low scores, but only for females. Additionally for females, high levels of extraversion and conscientiousness were also associated with more psychological aggression in study 2.

### Forensic Samples

Three studies used forensic samples in their studies. Study 9 recruited participants from three areas of Poland who had proceedings launched against them for, or had been convicted of, cruelty toward family members. Participants in study 10 were outpatients convicted of domestic violence with a comparison group of outpatients who were convicted of non-domestic and non-sexual violence. Study 11 recruited participants from IPV therapy programs. Studies 9 and 10 did not distinguish between IPV type (physical/psychological/sexual). Both studies compared the personality traits of their IPV participants to normal groups. Study 10 used a norm group obtained by Hoekstra et al. (1996), which was based on a general population sample, and also had a comparison group of generally violent offenders. In comparison with the norm group, the IPV perpetrators scored significantly higher on neuroticism, mirroring the results found in the general population samples in the studies above. The generally violent group in the study also scored significantly lower than the norm for conscientiousness and agreeableness as well as higher on neuroticism. Study 10 found no significant differences between generally violent and IPV offenders on any personality traits other than agreeableness while controlling for age. IPV offenders were more agreeable than generally violent offenders. Study 9 compared IPV offenders to the norm groups for the Revised NEO Personality Inventory (Costa & McCrae, 2008) and found there were no significant differences in any of the personality traits.

The main aim of study 11 was not to identify the personality traits of IPV offenders; rather, it was to investigate whether personality traits affect behavior change. However, the study produced some findings that contribute to the overall picture of the personality traits of IPV offenders. Study 11 reported that at the end of treatment for IPV, offenders who scored higher on conscientiousness reported significantly lower psychological, physical, and sexual IPV. Perpetrators who were high scorers on neuroticism demonstrated significant increases in psychological, physical, and sexual aggression compared to low scorers who demonstrated a reduction in the use of violence. Those who scored low on openness to experience, agreeableness, and conscientiousness showed an increase in physical IPV, while high scores demonstrated a noticeable reduction. Similarly, those who scored lower on those traits, in addition to extraversion, appeared to report an increase in sexual coercion.

## Discussion

Within general population studies, the perpetration of both physical and psychological violence demonstrates a link with high levels of neuroticism; however, the strengths of the relationships using correlational analysis were not high. Yet, the results of the studies were consistent despite the use of different analysis methods, with regressions having the additional benefit of being able to control confounding variables. Further, two studies (four and eight) used methods to measure IPV that have not been validated. The lack of validation and the inability to replicate the method could lead to unreliable results. While study 8 found results consistent with the other general population studies, study 4 found a contradictory result for female IPV, reporting it was negatively related to neuroticism. This could be a result of utilizing an untested measurement tool for IPV.

Some studies identified a role for agreeableness; however, this was not universal. The findings for sexual aggression were mixed although only two studies specifically investigated this type of aggression; therefore, more research is needed before any conclusions can be drawn. There were only three studies on forensic populations and the results from these were not conclusive. One study found no differences in personality traits between IPV perpetrators and the comparison group, while another reported that IPV perpetrators scored higher on neuroticism than the norm, as suggested by general population studies. However, it is noted that generally violent offenders also scored highly on neuroticism, as well as scoring lower on conscientiousness and agreeableness. Study 11 demonstrates that, within IPV perpetrators, there are both low and high scores on each domain and that these personality traits can affect treatment outcomes. Being able to split IPV perpetrators into two groups to measure treatment response could suggest that there is not one personality profile for this offender type. However, the results from the general population studies suggest the role of neuroticism cannot be ignored.

Neurotic individuals experience greater distress in response to stress, cannot cope adequately (Vollrath & Torgerson, 2000), and use less adaptive coping strategies (Cimbolic Gunthert et al., 1999). Emotional instability is linked with the use of emotional coping, which is less effective than problem-focused coping and is related to high levels of aggression (Carlo et al., 2012). Anger reactivity and hostility result from high levels of neuroticism (Decuyper et al., 2011). Research also suggests that marital functioning can be predicted by neuroticism (Bartholomew et al., 2015). Relationship satisfaction has consistently been linked to neuroticism in research, and it is suggested that this association is mediated by negative interactions between couples (Donnellan et al., 2004). A meta-analysis by Tehrani and Yamini (2020) reported that an avoidant conflict resolution style is commonly found in people who score highly on the neuroticism dimension. This type of conflict resolution technique is characterized by silence, withdrawing, and ignoring the partner’s concerns, and the outcome is noted to be “lose-lose” (Tehrani and Yamini, 2020).

Johnson (1995) identified two types of IPV; common couple violence (CCV) and intimate terrorism (IT). Whereas IT is used to control partners, CCV arises from poor conflict management that escalates to violence and is prevalent among both genders (Bartholomew et al., 2005; Johnson, 1995). Johnson and Ferraro (2000) identified that with the typologies identified by Holtzworth-Munroe and Stuart (1994), family-only offenders were likely to display CCV as the violence was primarily physical and the severity was low, whereas the “generally violent” and “dysphoric-borderline” groups displayed similarities to IT. This distinction between the two types of IPV was supported in a study of British samples by Graham-Kevan and Archer (2003), whose sample consisted of women residing at Women’s Aid shelters, males attending IPV treatment, and male prisoners, as well as their partners and male and female students.

The current review investigated personality traits in both forensic and general population samples, synthesizing the data separately. Comparing findings from clinical and general populations may not be helpful or useful as the aggression being investigated may be the result of two different types of IPV that are conceptually distinct (Graham-Kevan & Archer, 2003). CCV is primarily found among community samples, whereas IT is mostly found in clinical samples (Bartholomew et al., 2015) and utilized predominantly by males (Johnson, 1995; Graham-Kevan & Archer, 2003). IT, more recently termed “coercive control” (Stark, 2009), is described as a tactic within a wider pattern of behavior, motivated by the desire to exert control over one’s partner (Johnson & Ferraro, 2000). The consistency of this behavioral trend could suggest coercive control is not an emotional response that is likely to be displayed by an individual who scores highly on the neuroticism scale. This could explain the mixed results from the forensic sample studies included in the current systematic review, in which study 9 found convicted IPV offenders’ personality traits were no different from the norm, and study 11 was able to identify both high and low neuroticism scorers in his sample of IPV offenders receiving treatment. Only study 10 found that IPV perpetrators scored higher than the norm on neuroticism; however, it is also noted that generally violent offenders also scored higher. There appears to be little research pinpointing what might drive a perpetrator to coercively control their partner, although Johnson (2008) notes both education level and childhood family dynamics are risk markers.

The most common method for measuring IPV in the current study was through the use of self-report surveys. All eight general population studies used this method, as well as study 11. There are two main problems with this method: context and reliability. First, measuring IPV perpetration by asking about the frequency with which certain behaviors are committed does not allow for the context in which the act happened to be assessed. The motivation for violence varies and can include self-defense, emotional response, and control, but this cannot be established by the use of measures such as the CTS (Graham-Kevan & Archer, 2003). This makes it difficult to distinguish between the types of violence committed (CCV/IT) and could result in inappropriate responses and misleading statistics. A similar problem could occur by the focus on only physical violence (Graham-Kevan & Archer, 2003). Two of the three studies with forensic samples did not break down IPV into the different versions (physical/psychological/sexual) to examine links with personality traits and half of the general population studies solely looked at physical IPV. Only two studies (2 and 11) examined all three IPV versions.

Second, self-reporting allows for unreliable measurements of IPV and, subsequently, bias (Graham-Kevan & Archer, 2003). Both victims and perpetrators may underreport violence (Brousseau et al., 2011). This could be a result of shame or worries about police involvement. While there is some suggestion that underreporting of perpetration occurs by both men and women (Varley Thornton et al., 2010), it appears that this is more common in male perpetrators (Graham-Kevan & Archer, 2003; Varley Thornton et al., 2010). However, the results of study 6 suggest males are more forthcoming with reporting the perpetration of sexual coercion than their female partners are at reporting victimization. This underreporting could affect the validity of the results in the current studies.

A similar issue occurs with the measurement of personality traits. The FFM measures in the current studies were self-report questionnaires, which could allow for social desirability. Particularly in relation to forensic samples, and offenders, there is the belief, held by researchers and professionals alike, that criminals are likely to manipulate responses on self-report measures (Mills et al., 2003). This would then diminish the capability to link offending and personality traits (Wiebe, 2004). In future studies, this could be protected by collecting personality trait ratings on the participants from their partners or other close relations. Partner ratings tend to correspond highly with self-ratings (Furler et al., 2014), suggesting accuracy, and may be a useful addition to self-reports on personality measures (Cundiff et al., 2012). Additionally, the studies in the current review explore the traits in isolation; however, individual traits likely interact with each other to lead to behavior. Examining different traits in combination, for example high neuroticism with low agreeableness, to identify specific personality profiles could provide more usable information on personality features that contribute to violence to highlight areas for intervention.

The results of the current review are limited in application to forensic populations, due to the small number of studies available. An adherence to one model of personality could go some way to explain this. Several studies that examined personality characteristics were excluded because they did not measure traits in line with the FFM. The inclusion of wider measures and/or models of personality could increase the number of studies available for analysis. However, the convergent validity of different personality assessment tools would need comprehensive investigation for comparisons between studies to be made.

The results may also be affected by the sampling in the studies selected, as well at the inclusion criteria. Only studies written in English were included, which may have restricted the population to European and American participants, and as such the results may only be applicable to perpetrators from these countries. Further, not all studies reported on the ethnic make-up of their samples, while the ones that did had a strong white majority so it is not clear that the results can be applied to all ethnicities. The exception is study 5, in which only 60% of the sample was white. It is noted that all bar one of the community studies samples included both male and female perpetrators, as well as one of the three studies utilizing a forensic sample, suggesting the findings may apply to both genders. Findings are, however, unlikely to apply to non-heterosexual participants. Although two studies do report including participants in homosexual relationships, four studies include only heterosexual participants, while several studies do not report on participant sexuality. These factors limit the applicability of the findings.

## Conclusion

In conclusion, the evidence is supportive of neuroticism playing a role in the perpetration of violence within general population samples. This could be the result of increased reactivity to distress when interpersonal conflicts arise. This type of violence could be considered CCV. A second type of violence is coercive control, which is motivated by control and is less likely to be related to emotional instability and is found more commonly in forensic samples. This may explain why the current review found mixed results on the prevalence of neuroticism in forensic samples. Further research with forensic and more diverse samples is required to draw firm conclusions. Future studies should consider collecting third-party ratings of participants’ personality traits and investigate the impact of personality/neuroticism on different types of violence.

**Table:** Summary of Critical Findings of Each Personality Trait on IPV Perpetration. The Review Included 11 Studies.

Personality Trait	Critical Findings
Openness	Examined in seven studies. Two studies found a relationship; however, their findings were contradictory.
Conscientiousness	Examined in eight studies. Three studies found a negative relationship, while one found a positive relationship.
Extraversion	Examined in seven studies. Two studies found a positive relationship; one found a negative relationship.
Agreeableness	Examined in eight studies. Four studies found a negative relationship; one found a positive relationship.
Neuroticism	Examined in 11 studies. Nine studies found a positive relationship; one found a negative relationship.

**Table:** Summary of Critical Findings of Each Personality Trait on IPV Perpetration. The Review Included 11 Studies.Summary of Implications of the Review for Practice, Policy, and Research.

Practice	Identifying the factors contributing to the perpetration of violence can highlight the areas of focus for intervention programs. The results of this review suggest that conflict resolution skills and emotional management interventions could reduce the prevalence of common couple violence.
Policy	Understanding the context of the violence and the factors that predispose someone to violence could affect policies relating to the punishment of perpetrators and policies regarding early intervention, such as teaching emotion regulation and conflict resolution skills in school.
Research	This review highlights that further research into the personality traits of forensic samples who have perpetrated Intimate Partner Violence may be required to highlight any differences in community and clinical samples in personality traits. Future research may also explore other models and measures of personality
